# Low fish meal diet supplemented with probiotics ameliorates intestinal barrier and immunological function of *Macrobrachium rosenbergii via* the targeted modulation of gut microbes and derived secondary metabolites

**DOI:** 10.3389/fimmu.2022.1074399

**Published:** 2022-11-17

**Authors:** Xiaochuan Zheng, Bo Liu, Ning Wang, Jie Yang, Qunlan Zhou, Cunxin Sun, Yongfeng Zhao

**Affiliations:** ^1^ Key Laboratory for Genetic Breeding of Aquatic Animals and Aquaculture Biology, Freshwater Fisheries Research Center (FFRC), Chinese Academy of Fishery Sciences (CAFS), Wuxi, China; ^2^ Wuxi Fisheries College, Nanjing Agricultural University, Wuxi, China

**Keywords:** *Macrobrachium rosenbergii*, cottonseed protein concentrate, bacillus coagulans, fish meal, intestinal microbial, secondary metabolites

## Abstract

The unsuitable substitution ratio of fish meal by plant protein will reshape the intestinal microbial composition and intestine immunity. However, previous studies were mostly limited to investigating how different feed or probiotics characterized the microbial composition but ignored the biological interactions between bacteria and host physiology through secondary metabolites. Therefore, this study integrates the apparent indicators monitoring, 16S rDNA sequencing, and metabonomics to systematically investigate the effects of cottonseed protein concentrate (CPC) substitution of fish meal and *Bacillus coagulans* intervention on gut microbes, secondary metabolites, and intestinal immunity of *Macrobrachium rosenbergii*. Prawns were fed with three diets for 70 days: HF diets contained 25% fish meal, CPC in LF diets were replaced with 10% fish meal, and LF diets supplemented with 2 × 10^8^ CFU/g diet *B. coagulans* were designated as BC diets. Results showed that CPC substitution induced a significant decrease in digestive enzyme activities (trypsin and lipase) and gut barrier protein *PT-1* expression and a significant increase in γ-GT enzyme activity and inflammatory-related factors (*Relish* and *Toll*) expression. *B. coagulans* treatment mitigated the negative changes of the above indicators. Meanwhile, it significantly improved the expression levels of the barrier factor *PT-1*, the reparative cytokine *IL-22*, and *Cu/Zn-SOD*. CPC substitution resulted in a remarkable downregulated abundance of Firmicutes phyla, *Flavobacterium* spp., and *Bacillus* spp. *B. coagulans* treatment induced the callback of Firmicutes abundance and improved the relative abundance of *Sphingomonas*, *Bacillus*, and *Ralstonia*. Functional prediction indicated that CPC substitution resulted in elevated potential pathogenicity of microbial flora, and *B. coagulans* reduces the pathogenesis risk. Pearson’s correlation analysis established a significant positive correlation between differential genera (*Sphingomonas*, *Bacillus*, and *Ralstonia*) and secondary metabolites (including sphingosine, dehydrophytosphingosine, amino acid metabolites, etc.). Meanwhile, the latter were significantly associated with intestinal immunoregulation-related genes (*Cu/Zn-SOD*, *IL-22*, *PT-1*, *Toll*, and *Relish*). This study indicated that *B. coagulans* could mediate specific gut microbes and the combined action of multiple functional secondary metabolites to affect intestinal barrier function, digestion, and inflammation. Our study revealed the decisive role of gut microbes and derived secondary metabolites in the model of dietary composition-induced intestinal injury and probiotic treatment from a new perspective.

## Introduction

As a high-quality protein source, fish meal is widely used in aquafeeds, especially crustacean feeds, thanks to its good palatability and balanced nutritional composition (1). Due to the rapid development of aquaculture and the depletion of wild capture fisheries resources, the feed cost is gradually increasing, and the sustainable development of aquaculture is being seriously challenged (2). Therefore, one of the solutions is using more widely available and inexpensive plant proteins to partially or entirely replace fish meal in aquafeeds. In recent years, aquatic nutritionists have been conducting a series of studies on substituting fish meal with plant proteins (3).

However, with the in-depth study, plant protein substitution problems have gradually come to the fore. The excessive substitution of plant protein for fish meal leads to dysbacteriosis, impaired intestinal barrier function, reduced digestion and absorption of nutrients, and intestinal inflammation in aquatic animals. These side effects are attributed to the inherent anti-nutritional factors and unbalanced nutritional composition, which have received immense attention across aquaculture nutrition. For example, Miao et al. found that different dietary soybean meal substitutions for defatted fish meal resulted in a decrease in the relative abundance of beneficial intestinal bacteria and an increase in the abundance of conditionally pathogenic bacteria (4).

Persistent elevation of the substitutions proportion led to a significant upregulation of intestinal mucosal inflammatory factor expression (4). Zhang et al. reported that a 20% soybean meal substitution (basal fish meal level of 50%) induced intestinal mucosal barrier injury, downregulated immunization parameters, and enteritis of juvenile grouper (5). With the upgrading of the processing industry, the palatability and nutritional value of cottonseed protein concentrate obtained by low-temperature extraction, dephenolization, and moderate desugarization have significantly improved. Recent reports indicated that cottonseed protein concentrate could replace fish meal and soybean protein with a higher level, which is currently a preferable alternative (6). However, high levels of cottonseed protein concentrate (CPC) substitution will still cause oxidative damage and intestinal barrier damage in aquatic animals. Fu et al. (7) reported that substituting low-gossypol cottonseed meal for more than 20% fish meal gradually aggravated the intestinal barrier function damage of juvenile golden pompano (*Trachinotus ovatus*). The phenotypes appeared as a suppressed expression of tight junction and antioxidant-related genes and an intensified inflammation response (7).

Recently, probiotic bacteria alleviating the adverse effects of aquatic animals induced by plant protein substitution is arousing great interest. Studies on zebrafish (8) and bullfrog (*Lithobates catesbeianus*) (9) have shown that probiotics can improve the growth inhibition, metabolic disorder, intestinal microdysbiosis, and intestinal inflammation of aquatic animals caused by fish meal replacement. As such, increasingly comprehensive probiotic solutions for fish meal substitution to improve the host’s gut health and stress resistance are being proposed in the aquaculture industry. The probiotic *Bacillus coagulans* is a Gram-positive lactic acid-producing, spore-forming bacterial species. Recent studies have shown that *B. coagulans* GBI-30, 6086 could enhance plant protein digestion, absorption, and amino acid bioavailability (10, 11). Moreover, *B. coagulans* could promote the intestinal health of aquatic animals by strengthening the gut barriers, relieving oxidative stress, and modulating the intestinal microflora (12). Given this, this study chose *B. coagulans* as a regulator for mitigating the side effects of plant protein replacement fish meal.

Notably, recent medical studies have provided us with a new perspective: the regulation essence of host metabolic diseases and inflammatory diseases by probiotics, prebiotics, medicine, and other regulators is *via* the modulation of secondary metabolites, such as secondary bile acids (13), short-chain fatty acids (14), and branched-chain amino acids (15). These microbiota-derived metabolites signal to host immune and metabolism organs by ligand–receptor interaction, enabling the implementation of microbe–host communication (16). However, previous studies were mostly limited to investigating how different probiotics or feed types characterized intestinal microbial composition. Alternatively, it only evaluated the variations of growth performance and host physiological index but ignored the biological interactions between intestinal bacteria and host, i.e., the functions of bacterial exo-metabolites.

Therefore, our study evaluated the effects of *B. coagulans* on intestinal microbial composition and functional secondary metabolites related to the gut health of *Macrobrachium rosenbergii* based on dietary fish meal substitution treatment. Above all, we provided new complementary insights combining 16S flora sequencing profiling with metabolomics datasets of intestinal contents, aimed to reveal their interactions with host gut immune barrier function and interpret the mechanism of probiotics in alleviating the adverse effects of plant protein substitution.

## Materials and methods

### Ethics statement and prawn management

The use of prawns in this study was approved by the Animal Care and Use Committee of the Committee on the Ethics of Animal Experiments of Freshwater Fisheries Research Center. *M. rosenbergii* was obtained from the Freshwater Fisheries Research Center breeding base at the Chinese Academy of Fishery Science. After 15 days of domestication, 600 healthy prawns with a uniform initial weight (0.28 ± 0.02 g) were randomly assigned to three treatment groups with five replicates in each treatment and 40 individuals per replicate bucket (150-cm inner diameter, 45-cm water depth). The three experimental diets were fed at a rate of 5%–10% of body weight at 9:00, 13:00, and 18:00 daily. The feeding amount was observed and timely adjusted based on the amount of residual bait after 30 min, and the residual bait and feces were sucked out by siphoning prior to feeding. Prawns’ mortality was recorded daily. Daily continuous aeration was performed to ensure adequate dissolved oxygen (≥6 mg/ml). Water quality was measured once a week, and the water temperature was 28°C–32°C, nitrite ≤ 0.02 mg/L, ammonia ≤ 0.2 mg/L, pH = 7.0–8.5, and the trial period was 70 days.

### Experimental design, diet preparation, and growth assessment

The group containing 25% fish meal as the positive control group was designated as the HF group. The negative control group replaced 10% fish meal with CPC and was designated the LF group. Subsequently, *B. coagulans* JSSW-LA-07-1 was added to the LF diet at 2 × 10^8^ CFU/g diet under sterilized conditions and was designated as the BC group. The additional amount of *B. coagulans* JSSW-LA-07-1 was referred to in previous reports (17, 18). The strain was kept by the China General Microbiological Culture Collection Center (CGMCC No. 10182). The ingredients and proximal composition of the control groups are shown in **Table 1**. All raw materials were finely ground and passed through a 60-mesh sieve. Premix additives and raw materials were weighed accurately according to the formula, mixed step by step, and then squeezed into strips by a twin-screw extruder with a 1-mm aperture.

**Table 1:** Ingredients and proximate analysis of HF diets and LF diets.

Ingredients	HF	LF
	Concentration (% dry matter)	
Fish meal	25	15
Cottonseed protein concentrate		10
Chellocken meal	3	3
Poultry by-product powder	2	2
Squid paste	3	3
Shrimp meal	4	4
Soybean meal	19	19
Rapeseed meal	15.5	15.5
α-Starch	20.5	19.9
Fish oil:soybean oil	2	2.6
Lecithin powder	1	1
Cholesterol	0.3	0.3
Ecdysterone	0.01	0.01
Choline chloride	1	1
Premix^a^	1	1
Bentonite	0.69	0.69
Monocalcium phosphate	2	2
Proximate analysis		
Moisture	10.46	10.61
Crude protein	40.14	40.3
Ether extracts	5.98	5.42
Ash	11.19	12.1

a Premix supplied the following vitamins (IU or mg/kg) and minerals (g/kg): vitamin A, 25,000 IU; vitamin D3, 20,000 IU; vitamin E, 200 mg; vitamin K3, 20 mg; thiamin, 40 mg; riboflavin, 50 mg; calcium pantothenate, 100 mg; pyridoxine HCl, 40 mg; cyanocobalamin, 0.2 mg; biotin, 6 mg; folic acid, 20 mg; niacin, 200 mg; inositol, 1,000 mg; vitamin C, 2,000 mg; choline, 2,000 mg; calcium biphosphate, 20 g; sodium chloride, 2.6 g; potassium chloride, 5 g; magnesium sulfate, 2 g; ferrous sulfate, 0.9 g; zinc sulfate, 0.06 g; cupric sulfate, 0.02 g; manganese sulfate, 0.03 g; sodium selenate, 0.02 g; cobalt chloride, 0.05 g; potassium iodide, 0.004 g.

After a 70-day feeding trial, the survival rate and the weight of prawns in each group were recorded. The weight gain rate, specific growth rate, and feed conversion ratio were calculated in the following formulas:


Weight gain rate(WGR,%)=(Final weight−Initial weight)/initial weight×100

Specific growth rate(SGR,%/day)=(Ln final weight−Ln initial weight)/days×100

Feed conversion ratio(FCR)=Dry feed intake(g)/weight gain(g)

### Sample collection, RNA extraction, and DNA extraction

Hemolymph was collected using sterile 1-ml syringes from the pericardial cavity, mixing 1:1 with precooling anticoagulant solution, and immediately centrifuged at 8,000 r/min, 4°C for 10 min. The supernatant was collected and stored at −20°C before measuring antioxidant indicators. Part of the intestine was dissected aseptically and stored at −20°C for subsequent biochemical analysis. The remaining intestine was dissected aseptically, quick-frozen in liquid nitrogen, and stored at −80°C for RNA isolation and PCR analysis. The total RNA of intestine tissues was isolated using TRIzol (TaKaRa Biomedical Technology Co., Ltd., Beijing, China). RNA integrity was tested using 1% agarose gel electrophoresis. RNA concentration and purity were determined based on OD 260/280 readings (ratio > 1.8) using a NanoDrop ND-1000 UV Spectrophotometer (NanoDrop Technologies Co., Ltd., Wilmington, DE, USA). In addition, the adequate intestinal contents were collected aseptically and stored in a −80°C freezer for subsequent 16S rDNA sequencing and metabolomics analysis. Microbial DNA was extracted from the samples following the manufacturer’s protocol using the E.Z.N.A.^®^ Soil DNA Kit (Omega Bio-Tek, Norcross, GA, USA). The integrity of the extracted DNA was measured using a NanoDrop ND2000 spectrophotometer (Thermo Scientific, Waltham, MA, USA) and 1% agarose gel electrophoresis. Concentrations of isolated DNA were measured using a Quant-iT PicoGreen dsDNA Assay kit (Invitrogen, Carlsbad, CA, USA) and a fluorometer and diluted to 20 ng/μl. Samples were stored at −20°C until sequencing.

### Detection of biochemical indicators in hemolymph and intestine

The content of malondialdehyde (MDA) (A003-1-2) and the antioxidant enzymatic activities of catalase (CAT) (A00-1-1) and total superoxide dismutase (T-SOD) (A001-3-2) in hemolymph were detected and quantitated by using the commercial kits purchased from Nanjing Jiancheng Institute of Bioengineering (Nanjing, China), according to the manufacturer’s instructions. Approximately 0.1 g of intestinal tissue was minced and homogenized in ice-cold 0.86% stroke-physiological saline solution (w/v, 1:9) using an Ultra-Turrax homogenizer (Tekmar Co., Cincinnati, OH, USA) and centrifuged at 4,000 r/min at 4°C for 10 min to obtain the supernatant for further analysis. The digestive enzymes and brush border enzyme indicators including trypsin, lipase, amylase, and γ-GT were determined using commercial assay kits (A080-2-2, A054-2-1, C016-1-1, and C017-2-1, Nanjing Jiancheng Bioengineering Institute, Nanjing, China) according to the manufacturer’s protocols.

### Quantitative real-time PCR

The extracted total RNA from the intestine was synthesized using a Perfect Real Time SYBR Prime Script TM RT Reagent Kit (TaKaRa Biotechnology, Dalian, China) as per the manufacturer’s instructions. The reaction parameters of RTqPCR are as follows: pre-run at 95°C for 15 min, followed by 40 cycles of denaturation at 95°C for 15 s, and a 60°C annealing step for 34 s. The reaction mixture comprised 2 µl of cDNA, 0.4 µl of forward primer, 0.4 µl of reverse primer, 10 µl of SYBR Premix ExTaqTM (TaKaRa, Dalian, Liaoning, China), 0.4 µl of ROX Reference Dye (TaKaRa, Dalian, Liaoning, China), and 6.8 µl of double-distilled water. *β-Actin* gene was selected as a reference gene (19), and each sample was tested in triplicate. Primers were designed online (NCBI, Bethesda, MA, USA) (**Table 2**). Notably, the CDS sequences used in the primer design of some genes were obtained from our laboratory’s database of the hepatopancreas transcriptome sequencing of *M. rosenbergii*. The results were calculated by the 2^−ΔΔCt^ method, and the gene-specific primers were synthesized by Shanghai Generay Biotech Co., Ltd. (Shanghai, China).

**Table 2:** Primer sequences for real-time PCR.

Gene	Primers	Sequence 5′–3′	Product length (bp)	Reference
*Toll*	F	TTCGTGACTTGTCGGCTCTC	145	KX610955.1
	R	GCAGTTGTTGAAGGCATCGG		
*Relish*	F	GATGAGCCTTCAGTGCCAGA	149	KR827675.1
	R	CCAGGTGACGCCATGTATCA		
*IL-22*	F	ACGAGCTGCGATCCAGTAAG	101	Cluster-21039.10593
	R	GCAACGCACTGCTCCTTAAC		
*Cu-Zn-SOD*	F	AGAGCAGTTGTAGGCCGAAG	116	EU077527.1
	R	GTGCAGCAAGCCAATCTAGC		
*PT-1*	F	TTGCTTGGTCAGTCTCCTGC	106	Cluster-1871.0
	R	CTCTAAGGTCTGGGCCTGTC		
*β-Actin*	F	TCCGTAAGGACCTGTATGCC	101	AY651918.2
	R	TCGGGAGGTGCGATGATTTT		

### 16S rRNA sequencing of intestinal microorganisms

The target fragment library was prepared by PCR amplification of the V4–V5 region of the 16S rRNA gene. The universal primers 515 F (5′-GTGCCAGCMGCCGCGG-3′) and 907 R (5′-CCGTCAATTCMTTTRAGTTT-3′) were used to amplify the V4–V5 region of the 16S rRNA gene. PCR products were detected by 2% agarose gel electrophoresis, and target fragments were collected using the AxyPrep DNA Gel Extraction Kit. Amplicons of every sample were combined, purified, and quantified. Library construction and double-ended sequencing were conducted using the Illumina HiSeq platform to produce 2 × 250-bp paired-end reads. Raw fastq files were de-multiplexed and quality-filtered by removing the reads’ barcode and linker sequences and merging with the paired-end reads into a longer fragment. Then, reads that were truncated at any site from the beginning of the window to the 3′ terminus and received an average quality score<20 over a 6-bp sliding window, and truncated reads<100 bp were discarded. Mismatching primer sequences or ambiguous bases (Ns) exceeding 5% were removed from the downstream analyses, and reads that could not be assembled were discarded. FLASH (fast length adjustment of short reads, v1.2.8, http://ccb.jhu.edu/software/FLASH) was used for spliced paired-end sequences, and chimeric sequences were removed using VSEARCH (https://github.com/torognes/vsearch, v2.3.4). Finally, the operational taxonomic units (OTUs) were generated after denoising. Quality-filtered reads were clustered into OTUs following the criterion of more than or equal to 97% similarity. For detailed steps and parameters of 16S rRNA, refer to our previous description (20).

### Metabolomics analysis of intestinal contents

Intestinal contents samples were prepared from the prawns of the LF and BC groups. Each sample measuring 150 mg was accurately weighed and placed into the EP tube, added with 800 μl of pre-cooled 50% methanol–water (1:1, V:V), and grinded thoroughly in a tissue grinder (50 Hz, 5 min). After three centrifugations at 4°C, 2,500 *g*, 550 μl of supernatant was extracted and dried in the freeze dryer. Then, 600 μl methanol–water (1:9, V:V) was added to re-dissolve and vortex the residue and perform ultrasonic treatment, and finally, the supernatant was obtained. Meanwhile, 50 μl of supernatant from each sample was mixed to be the quality control (QC) sample, and all the specimens were stored at −80°C until liquid chromatography–tandem mass spectrometry (LC-MS/MS) analysis. The ACQUITY UPLC BEH C18 column (100 mm * 2.1 mm, 1.7 μm, Waters, Cheshire, UK, column temperature 50°C) was used for chromatographic separation, and the injection volume was 10 μl. The high-resolution tandem mass spectrometry Xevo G2-XS QTOF (Waters, UK) was used to collect the small molecules eluted from the column, under the positive (ES+) and negative ion modes (ES−). The raw data were imported into commercial software Progenesis QI (version 3.0.3) for peak alignment, peak extraction, and peak identification and data correction, and the mass charge ratio, retention time, and ion area of metabolites were obtained. Then, the metabolomics R software package-Metax was used to remove low-quality ions. The univariate and multivariate analyses were used to identify the differential metabolites with different abundances under different treatment conditions. The variable importance in projection (VIP) was calculated to assist in the screening of metabolic markers. This study used multivariate analysis of VIP values of the first two principal components of the partial least squares discriminant analysis (PLS-DA) model, combined with univariate analysis of fold change and *p*-value to screen the differential ions. The differential ions should simultaneously satisfy the following three conditions: 1) VIP ≥ 1, (2) fold change ≥1.2 or ≤0.8333, and 3) *p*-value< 0.05. The identification databases of metabolites included the Kyoto Encyclopedia of Genes and Genomes (KEGG), Human Metabolome Database (HMDB), and LIPID MAPS commercial databases. The differential metabolites were identified and screened out based on the fragment score, isotope similarity, mass error, and the pathway annotation results of the differential ions. Finally, the KEGG pathway analysis of different metabolites was performed. For detailed steps and parameters of metabolomics, refer to our previous description (21).

### Statistical analysis

The differences in growth performance parameters, biochemical indicators, and gene expression parameters were analyzed using one-way ANOVA and Tukey’s honestly significant difference (Tukey’s HSD) (SPSS 22.0 software, Chicago, IL, USA). Statistically significant was considered when *p*< 0.05. The above data were expressed as means ± SEMs, and before statistical analysis, all data were tested for the normality of distribution and homogeneity of variances with Levene’s test. The alpha diversity was calculated using QIIME software, including Chao1, observed species, Shannon index, and Simpson index. An independent-samples t-test was performed for all diversity indices and presented as mean ± standard deviation (SD). Statistically significant was considered when *p*< 0.05. The R package drew the principal coordinates analysis (PCoA) diagram. The microbial community and relative abundance values analysis were conducted using STAMP software. Mean differences were considered significant at *p*< 0.05. Furthermore, intestinal differential metabolite clusters were conducted using a Pearson’s correlation test with differential microbial genera abundance and intestinal gene richness. Tables were generated using Excel 2016, while figures were made using GraphPad Prism 8.

## Results

### Growth performance indicator determination

As shown in **Table 3**, the levels of growth performance indicators were not significantly different among the three groups; however, WGR, SGR, and SR clearly presented a trend of decreasing followed by increasing, and FCR showed contrary trends.

**Table 3:** Effect of cottonseed protein concentrate as a substitute for fish meal and *Bacillus coagulans* supplementation on the growth performance of *Macrobrachium rosenbergii*.

Group	FW (g)	WGR (%)	SGR (%/day)	FCR	SR (%)
HF	4.22 ± 0.28	1,521.85 ± 71.92	3.98 ± 0.06	1.63 ± 0.12	84.17 ± 3.63
LF	4.29 ± 0.16	1,489.40 ± 37.13	3.95 ± 0.03	1.69 ± 0.04	80.83 ± 1.67
LF+BC	4.93 ± 0.47	1,582.71 ± 134.00	4.02 ± 0.11	1.42 ± 0.18	86.67 ± 4.17

FW, final weight; WGR, weight gain rate; SGR, specific growth rate; FCR, feed conversion ratio; SR, survival rate.

### Analysis of hemolymph antioxidant indicators

**Figure 1:**
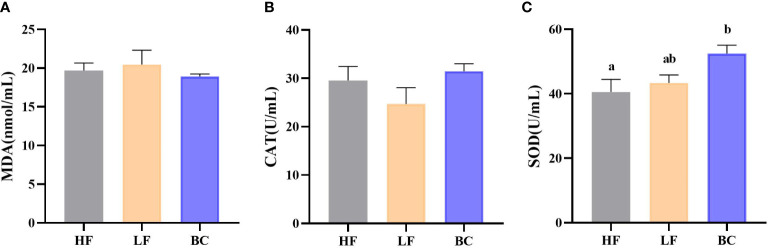
**(A–C)** Hemolymph antioxidant indicators. **(A)** Hemolymph MDA content; **(B)** Hemolymph CAT activity; **(C)** Hemolymph SOD activity.

**Figure 1** shows no significant difference in hemolymph MDA contents and CAT levels of prawns among these three groups. The hemolymph SOD activities of prawns were significantly increased in the BC group compared with the HF group.

### Intestinal digestive enzymes and brush border enzyme activity determination

**Figure 2:**
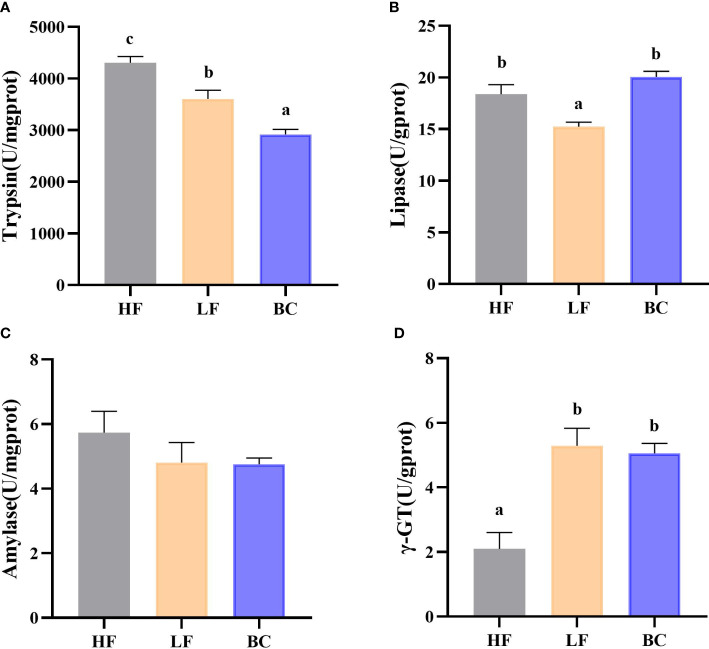
**(A–D)** Intestinal digestive enzymes and brush border enzyme activity. **(A)** Intestinal trypsin activities; **(B)** Intestinal lipase activities; **(C)** Intestinal amylase activities; **(D)** Intestinal γ-GT activities.

As shown in **Figure 2**, the trypsin activities of prawns were significantly decreased in the LF group compared with the HF group, and the lowest level was found in the BC group. No significant difference in lipase activity was found between the HF and BC groups; the activity in LF was significantly decreased compared with these two groups. There was no significant difference in amylase activities among these three groups. The γ-GT activities of prawns were considerably increased in the LF and BC groups compared with the HF group.

### Analysis of intestinal gene expression profile

**Figure 3:**
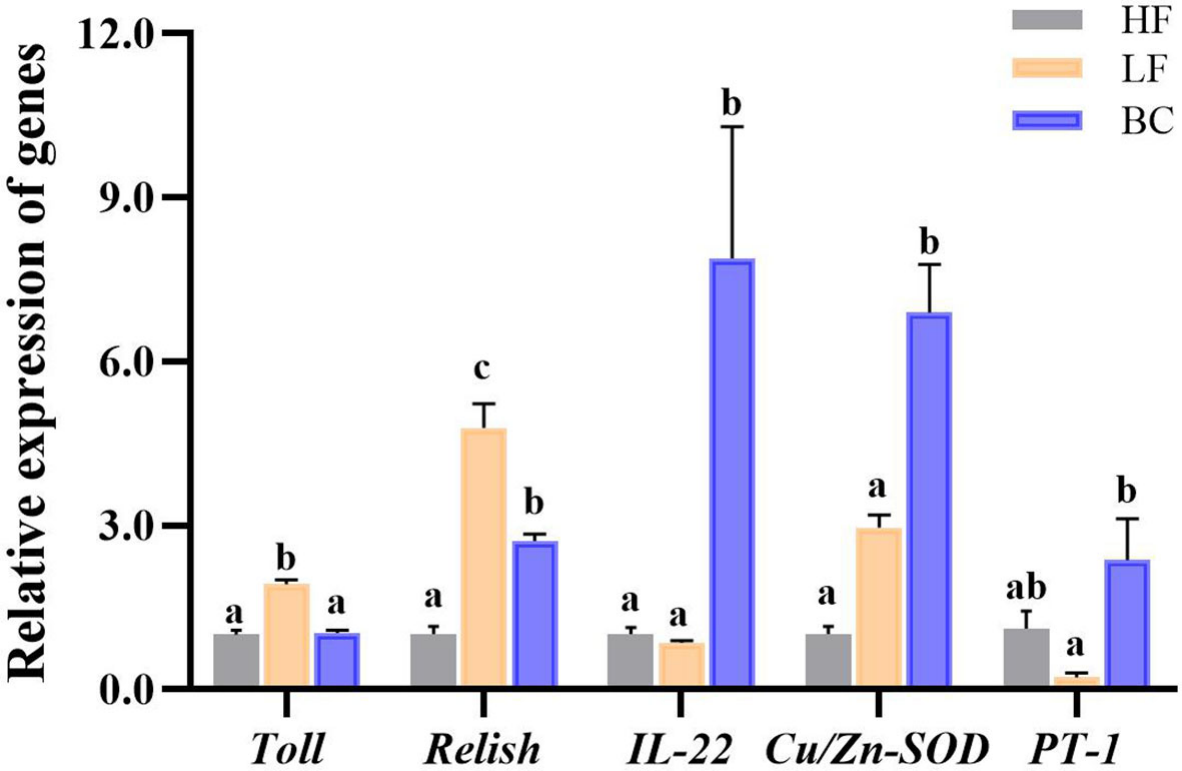
Intestinal gene expression profile. Different lowercase letters indicate significant differences (P < 0.05) between the three groups.

As shown in **Figure 3**, the *Toll* and *Relish* expression levels of prawns were significantly upregulated in the LF group compared with the HF group. BC intervention downregulated the *Toll* and *Relish* expression levels compared to the LF group. BC intervention significantly downregulated the expression of *Toll* to a level close to the HF group; however, the *Relish* expression level in the BC group was significantly higher than in the HF group. The *IL-22* and *Cu/Zn-SOD* expression levels of prawns were significantly upregulated in the BC group than the HF and LF groups, and no significant difference was found between the HF and LF groups. The *PT-1* expression in the BC group was significantly upregulated compared with the LF group, and there was no significant difference between the HF and LF groups.

### Community diversity analysis of prawns’ intestinal microflora

**Figure 4:**
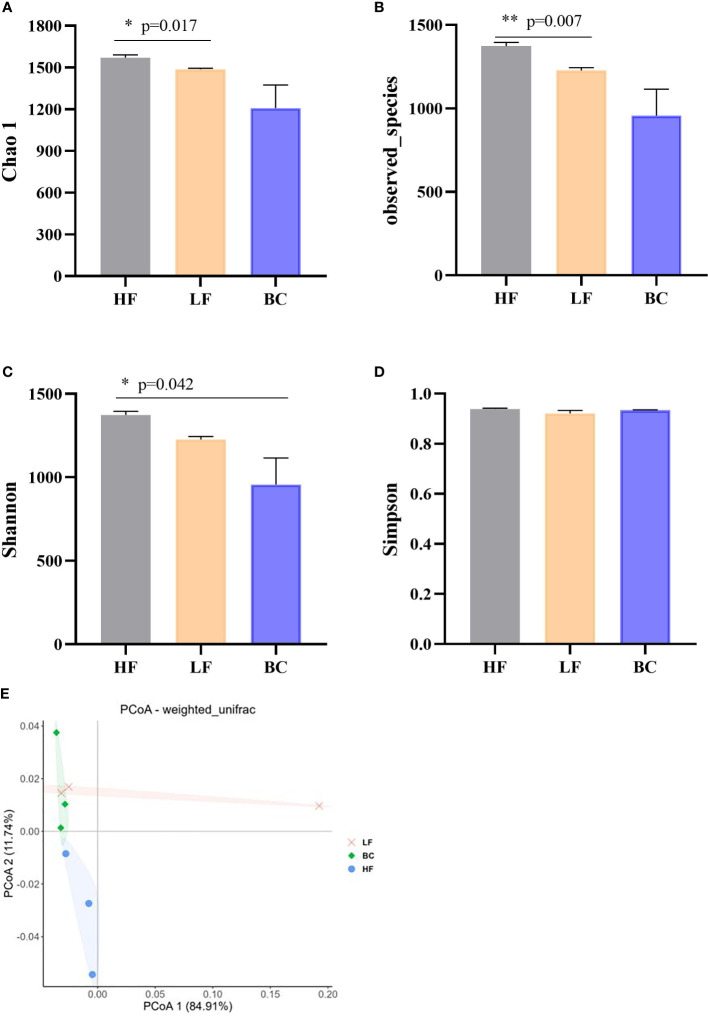
**(A–E)** Community diversity analysis and principal coordinates analysis (PCoA) of bacterial communities of *Macrobrachium rosenbergii* fed with different diets. * P < 0.05, ** P < 0.01.

This study obtained 2,682 OTUs from all samples. The Chao1, observed species, Shannon, and Simpson indices were calculated to evaluate the microbial community richness and diversity of prawns’ intestines. As shown in **Figure 4**, the Chao 1 and observed_species index of prawns’ intestinal microflora in HF were significantly higher than in LF prawns. There was no significant difference in Chao 1 and observed_species index between the BC group and the other two groups. The Shannon index of prawns’ intestinal microflora in the BC group was significantly lower than in the HF group. No significant difference was found in the Simpson index among these three groups. PCoA of bacterial communities of *M. rosenbergii* fed with three different diets showed that the LF group was distant from the HF group samples. In contrast, the BC group sample cluster intersected the LF and HF groups, shortening the distance from the HF group samples.

### Gut microbiota function prediction

**Figure 5:**
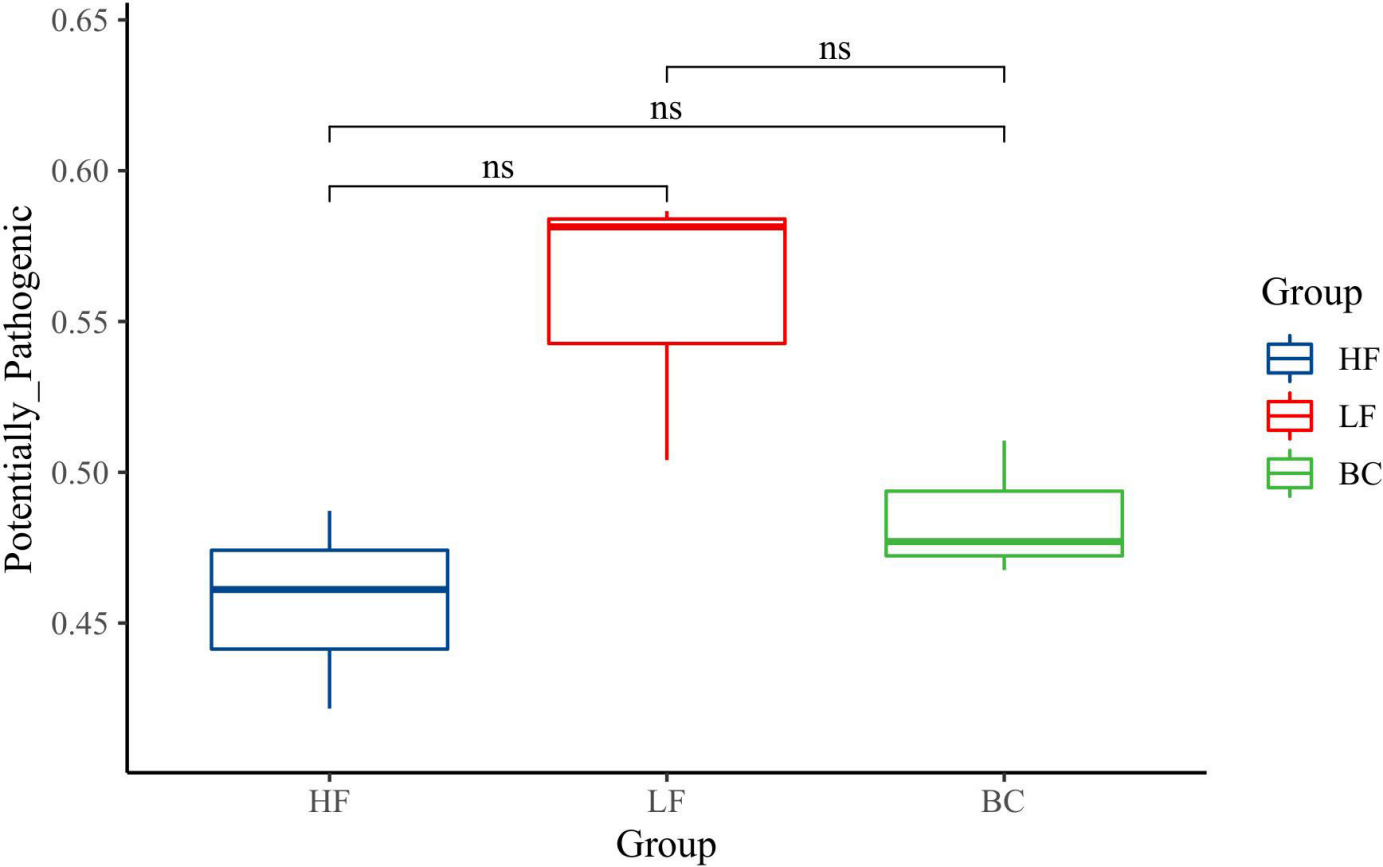
Gut microbiota function prediction. ns, P > 0.05.

The predicted_phenotypes of potentially_pathogenic by BugBase software are shown in **Figure 5**. The potentially_pathogenic of prawns in the LF group was higher than in the HF group, and the BC group’s predicted_phenotypes were decreased compared with the LF group. There was no significant difference among these three groups.

### Microbial community structure of *Macrobrachium rosenbergii* treated with different diets

**Figure 6:**
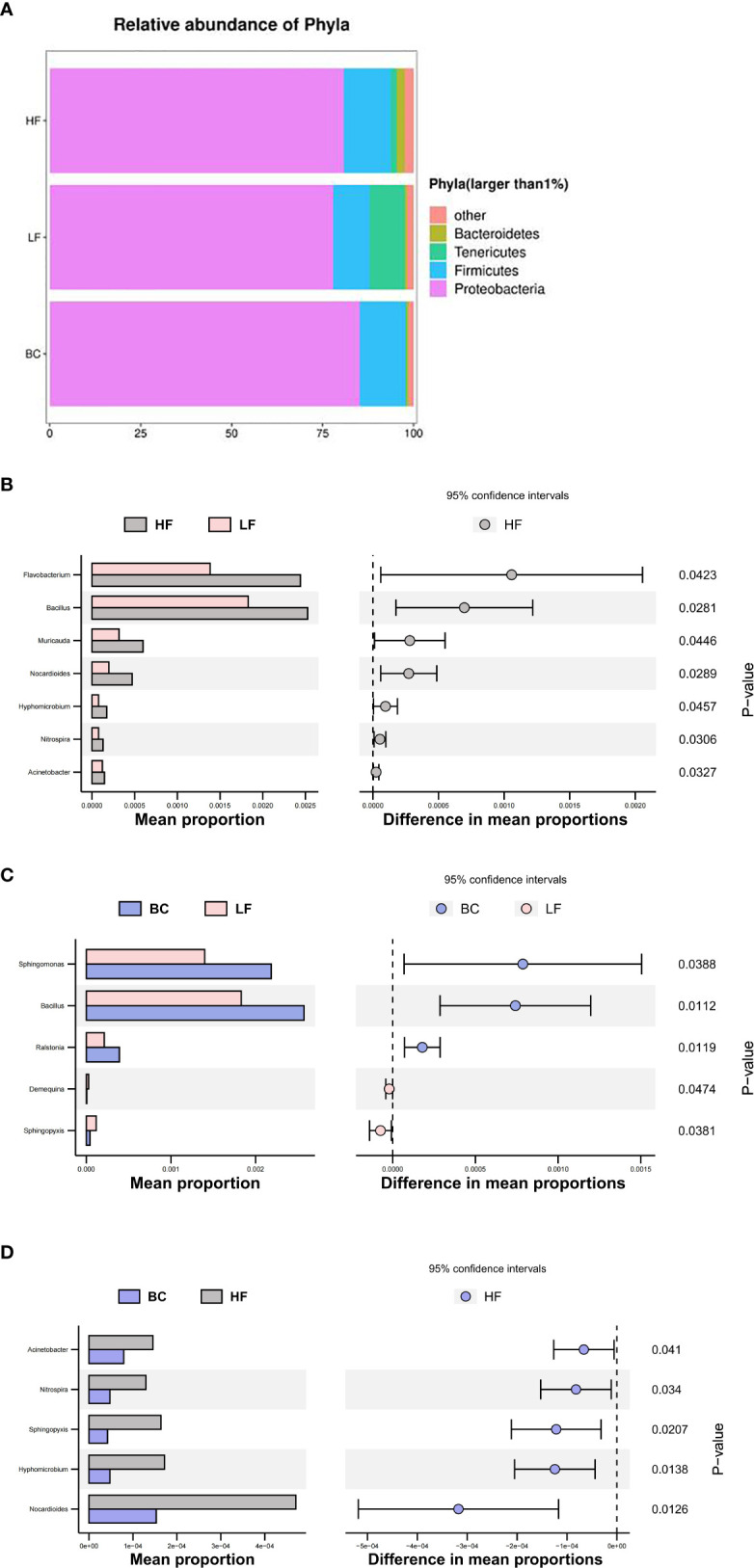
**(A–D)** Microbial community structure of *Macrobrachium rosenbergii* treated with different diets.

As shown in **Figure 6**, four predominant phyla (mean relative abundance >1%) were detected in *M. rosenbergii* samples, including Proteobacteria (81.33%), Firmicutes (11.84%), Tenericutes (3.85%), and Bacteroidetes (1.11%). Compared with the HF group, the mean relative abundance of Proteobacteria, Firmicutes and Bacteroidetes decreased in the LF group, while the Tenericutes increased. *B. coagulans* treatment adjusted the relative abundance of these changed genera to a level similar to that of the HF group, except for Bacteroidetes. At the genus level, compared with the HF group, the main differential bacteria in the LF group were *Flavobacterium*, *Bacillus*, *Muricauda*, *Nocardioides*, *Hyphomicrobium*, *Nitrospira*, and *Acinetobacter*, all of which showed decreased abundance. Compared with the LF group, the different main bacteria in the BC group were *Sphingomonas*, *Bacillus*, *Ralstonia*, *Demequina*, and *Sphingopyxis*, of which the abundance of the former was significantly upregulated, and the latter two were significantly decreased. Compared with the BC group, a higher abundance of *Nocardioides*, *Hyphomicrobium*, *Sphingopyxis*, *Nitrospira*, and *Acinetobacter* was found in the HF group.

### Analysis of the differential metabolites in fecal between LF and BC groups

The differential metabolites of intestinal flora between the LF and BC groups are listed in **Table 4**. Among them, 89 metabolites were significantly upregulated, and one metabolite was significantly downregulated in the BC group. The differential metabolites mainly included amino acids and amino acid derivatives, sphingolipids and other lipids, aromatic compounds, energy metabolism-related metabolites, secondary metabolism of plants, nucleotides, and nucleotide derivatives.

**Table 4:** The differential metabolites in fecal between LF and BC groups.

Metabolites	rt	Mass	MeanLF	MeanBC	Trend	VIP	*p*-Value	FC
**Amino acids and amino acid derivatives**								
l-Serine	389.699	106.050	1.437	3.838	Up	1.235	0.047	2.671
l-Lysine	532.865	147.112	3.324	19.025	Up	1.318	0.006	5.723
beta-Alanine	359.246	90.055	10.680	24.322	Up	1.243	0.024	2.277
d-Ornithine	547.123	133.097	0.386	1.065	Up	1.229	0.036	2.760
d-Proline	514.857	116.070	1.293	4.484	Up	1.316	0.010	3.467
Tryptophan	0.876	205.096	4.911	16.538	Up	1.242	0.018	1.717
5-Hydroxy-l-tryptophan	297.080	221.091	0.051	0.190	Up	1.217	0.039	3.701
l-Norleucine	279.448	130.086	255.886	427.238	Up	1.283	0.045	1.670
l-Valine	313.992	118.086	24.588	58.664	Up	1.250	0.037	2.386
l-Phenylalanine	273.762	166.085	35.455	102.182	Up	1.275	0.029	2.882
l-Tyrosine	316.869	182.080	8.767	33.059	Up	1.225	0.027	3.771
Prolylglycine	311.634	173.091	0.045	0.160	Up	1.236	0.019	3.531
l-Glutamic acid	411.255	148.059	10.094	24.526	Up	1.204	0.035	2.430
l-Asparagine	389.770	133.060	3.387	9.566	Up	1.228	0.029	2.825
Argininosuccinic acid	484.113	291.127	0.052	0.179	Up	1.230	0.028	3.430
Glutamyllysine	506.193	276.153	0.014	0.089	Up	1.331	0.001	6.526
Gamma-glutamyl ornithine	465.060	262.138	0.116	0.419	Up	1.316	0.005	3.601
Lysyl-Glycine	470.018	204.133	0.543	1.578	Up	1.251	0.021	2.906
Leucyl-Glycine	120.022	189.122	1.042	2.057	Up	1.174	0.043	1.974
Lysyl-Arginine	572.540	303.211	0.080	0.462	Up	1.332	0.003	5.748
Lysyl-Aspartate	482.117	262.138	0.022	0.093	Up	1.276	0.028	4.164
Aspartyl-Arginine	456.445	290.143	0.254	0.816	Up	1.282	0.021	3.215
Lysyl-Lysine	574.436	275.206	0.043	0.237	Up	1.344	0.000	5.507
Prolyl-Lysine	485.670	244.164	0.412	1.618	Up	1.247	0.020	3.924
Hydroxyprolyl-Arginine	380.695	288.165	0.094	0.160	Up	1.171	0.048	1.706
*N* ^6^-Acetyl-l-lysine	403.155	189.122	0.108	0.230	Up	1.195	0.036	2.125
Epsilon-(gamma-glutamyl)-lysine	342.282	114.091	0.426	0.164	down	1.294	0.002	0.385
Vinylacetylglycine	445.764	144.065	0.948	1.564	Up	1.215	0.035	1.650
*N*-Acetyldopamine	389.971	196.096	0.052	0.139	Up	1.206	0.035	2.658
5-Aminopentanal	268.421	102.091	0.855	1.795	Up	1.146	0.050	2.100
5-Aminopentanoic acid	285.672	118.086	153.482	232.481	Up	1.193	0.037	1.515
(3*S*,5*S*)-3,5-Diaminohexanoate	358.914	147.112	0.082	0.127	Up	1.221	0.031	1.543
**Sphingolipids**								
Sphinganine	126.829	302.303	0.150	0.483	Up	1.245	0.030	3.213
Dehydrophytosphingosine	150.057	316.283	2.173	4.968	Up	1.266	0.018	2.286
**Phospholipids**								
PC(15:0/15:0)	165.938	706.534	0.876	2.051	Up	1.219	0.044	2.341
PC(16:0/16:0)	143.618	734.567	0.113	0.247	Up	1.166	0.049	2.182
PC(16:1(9*Z*)/14:0)	165.938	704.517	0.871	1.784	Up	1.224	0.027	2.048
PC(16:1(9*Z*)/15:0)	165.148	718.532	0.430	1.172	Up	1.282	0.006	2.726
PC(18:1(11*Z*)/14:0)	163.671	732.548	7.850	16.581	Up	1.262	0.017	2.112
PC(16:1(9*Z*)/16:1(9*Z*))	164.080	730.535	2.463	5.134	Up	1.203	0.042	2.085
PC(18:2(9*Z*,12*Z*)/18:0)	158.697	786.596	11.337	23.771	Up	1.263	0.008	2.097
PC(15:0/18:2(9*Z*,12*Z*))	163.281	744.549	0.774	1.979	Up	1.248	0.015	2.557
PC(24:1(15*Z*)/14:1(9*Z*))	61.254	814.623	0.138	0.500	Up	1.257	0.012	3.630
PC(20:2(11*Z*,14*Z*)/15:0)	160.063	772.580	0.970	2.096	Up	1.263	0.013	2.162
PC(16:1(9*Z*)/P-18:1(11*Z*))	157.795	742.570	0.117	0.261	Up	1.303	0.009	2.221
PC(18:3(6*Z*,9*Z*,12*Z*)/15:0)	163.278	742.532	0.383	0.964	Up	1.277	0.010	2.517
PC(18:3(9*Z*,12*Z*,15*Z*)/14:0)	164.912	728.519	0.237	0.600	Up	1.246	0.041	2.536
PC(22:2(13*Z*,16*Z*)/16:1(9*Z*))	157.827	812.611	1.076	1.904	Up	1.194	0.042	1.769
PC(18:3(6*Z*,9*Z*,12*Z*)/18:1(11*Z*))	59.786	782.561	4.189	14.056	Up	1.185	0.028	3.355
PC(18:3(6*Z*,9*Z*,12*Z*)/P-18:1(11*Z*))	156.085	766.568	2.699	4.163	Up	1.169	0.046	1.542
PC(18:4(6*Z*,9*Z*,12*Z*,15*Z*)/18:1(11*Z*))	60.817	780.549	5.307	23.397	Up	1.270	0.010	4.409
PC(18:4(6*Z*,9*Z*,12*Z*,15*Z*)/P-18:1(11*Z*))	154.689	764.555	1.898	3.151	Up	1.242	0.013	1.661
PC(20:2(11*Z*,14*Z*)/20:4(5*Z*,8*Z*,11*Z*,14*Z*))	153.256	834.593	4.658	9.579	Up	1.230	0.044	2.056
PC(22:5(7*Z*,10*Z*,13*Z*,16*Z*,19*Z*)/20:1(11*Z*))	150.997	862.623	0.162	0.494	Up	1.290	0.009	3.048
PC(P-18:1(11*Z*)/22:5(4*Z*,7*Z*,10*Z*,13*Z*,16*Z*))	149.122	818.601	0.802	1.068	Up	1.237	0.042	1.332
PE(P-16:0/20:5(5*Z*,8*Z*,11*Z*,14*Z*,17*Z*))	155.152	722.508	1.542	2.651	Up	1.181	0.048	1.719
LysoPE(0:0/14:0)	224.850	426.258	0.013	0.048	Up	1.235	0.022	3.755
LysoPC(16:0)	212.374	496.336	6.094	25.253	Up	1.253	0.021	4.144
LysoPC(P-16:0)	205.967	480.341	0.194	0.479	Up	1.250	0.020	2.470
LysoPC(18:1(9*Z*))	209.520	522.353	14.907	66.989	Up	1.226	0.034	4.494
SM(d18:1/16:0)	200.851	703.569	0.195	0.937	Up	1.332	0.001	4.798
SM(d18:1/20:0)	197.867	759.630	0.029	0.118	Up	1.267	0.018	4.072
SM(d18:0/18:1(9*Z*))	198.900	731.601	0.113	0.611	Up	1.319	0.002	5.425
**Other lipids**								
(2*E*)-Decenoyl-ACP	532.921	130.085	1.731	8.939	Up	1.317	0.007	5.164
Phosphodimethylethanolamine	460.647	170.057	0.123	0.561	Up	1.312	0.003	4.561
5α-Cholesta-8,24-dien-3-one	31.273	383.328	0.201	0.341	Up	1.225	0.026	1.702
**Aromatic compounds**								
Quinone	223.123	109.028	0.106	0.386	Up	1.220	0.026	3.653
Phenol	316.869	95.049	0.194	0.763	Up	1.212	0.037	3.929
Parabanic acid	16.343	112.998	0.825	0.976	Up	1.495	0.012	1.183
Benzaldehyde	273.701	107.049	0.358	1.088	Up	1.291	0.022	3.037
4-Hydroxybenzaldehyde	316.993	123.044	1.072	4.121	Up	1.221	0.031	3.844
2-Phenylacetamide	184.356	128.143	11.688	20.587	Up	1.280	0.018	1.761
1*H*-Indole-3-carboxaldehyde	276.993	146.059	0.518	1.876	Up	1.190	0.028	3.619
1*H*-Indole-2,3-dione	47.215	148.038	0.163	0.464	Up	1.209	0.029	2.855
**Energy metabolism-related**								
*O*-Phosphotyrosine	492.769	262.049	0.271	1.058	Up	1.308	0.002	3.902
Isocitral	183.881	153.127	0.087	0.177	Up	1.147	0.041	2.034
3-Dehydroxycarnitine	390.707	146.117	31.835	78.692	Up	1.260	0.018	2.472
**Secondary metabolites of plants**								
7-Hydroxy-2-phenyl-4*H*-chromen-4-one	202.998	237.054	0.326	0.640	Up	1.569	0.011	1.961
Glycitein	145.862	307.060	0.154	0.317	Up	1.327	0.001	2.061
Luteolin 4′-sulfate	26.207	367.009	0.032	0.100	Up	1.318	0.001	3.154
Momordicinin	30.867	439.355	0.026	0.045	Up	1.311	0.002	1.708
Isoquinoline	137.025	130.064	0.099	0.269	Up	1.279	0.018	2.713
2-Propylpiperidine	184.356	128.143	11.688	20.587	Up	1.280	0.018	1.761
Pipericine	1.954	336.324	0.007	0.016	Up	1.139	0.044	2.296
**Nucleotides and nucleotide derivatives**								
8-Hydroxyguanine	309.359	168.051	0.253	0.403	Up	1.189	0.045	1.593
Pyrimidine	172.823	81.045	0.134	0.585	Up	1.259	0.043	4.361
3-Aminoisobutanoic acid	350.392	102.055	0.655	1.177	Up	1.500	0.027	1.796
Cyclic AMP	281.191	328.044	0.319	1.564	Up	1.461	0.041	4.903
3′-AMP	420.136	346.055	0.424	0.863	Up	1.330	0.041	0.662
Vitamin D3	31.910	385.343	0.164	0.326	Up	1.204	0.039	1.988

### Kyoto Encyclopedia of Genes and Genomes pathway enrichment analysis of differential metabolites between LF and BC groups

**Figure 7:**
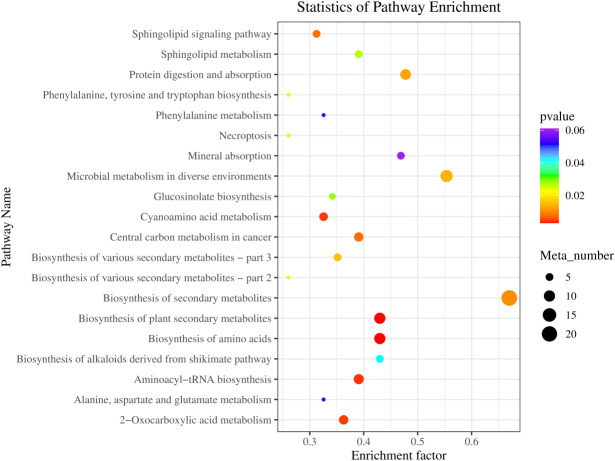
KEGG pathway enrichment of differential metabolites in fecal between LF and BC groups. Colors indicate enriched Q-value; the redder color represents a smaller Q-value. The size of the bubble indicates the number of metabolites mapped within a KEGG pathway. KEGG, Kyoto Encyclopedia of Genes and Genomes.

As shown in **Figure 7**, the top 20 KEGG pathways with significant enrichment are as follows: Secondary metabolite synthesis-related (Biosynthesis of secondary metabolites, Biosynthesis of plant secondary metabolites, Glucosinolate biosynthesis, Biosynthesis of alkaloids derived from shikimate pathway, Biosynthesis of various secondary metabolites-part 3, Biosynthesis of various secondary metabolites-part, Sphingolipids metabolism-related, Sphingolipid signaling pathway, and Sphingolipid metabolism), Amino acid synthesis, metabolism and transfer-related (Biosynthesis of amino acids; Aminoacyl-tRNA biosynthesis; Cyanoamino acid metabolism; Phenylalanine metabolism; Alanine, aspartate, and glutamate metabolism; Phenylalanine, tyrosine, and tryptophan biosynthesis), Microbial metabolism in diverse environments, Protein digestion and absorption, Central carbon metabolism in cancer, 2-Oxocarboxylic acid metabolism, Necroptosis, and Mineral absorption.

### Correlation analysis of intestinal microbes and metabolites

**Figure 8:**
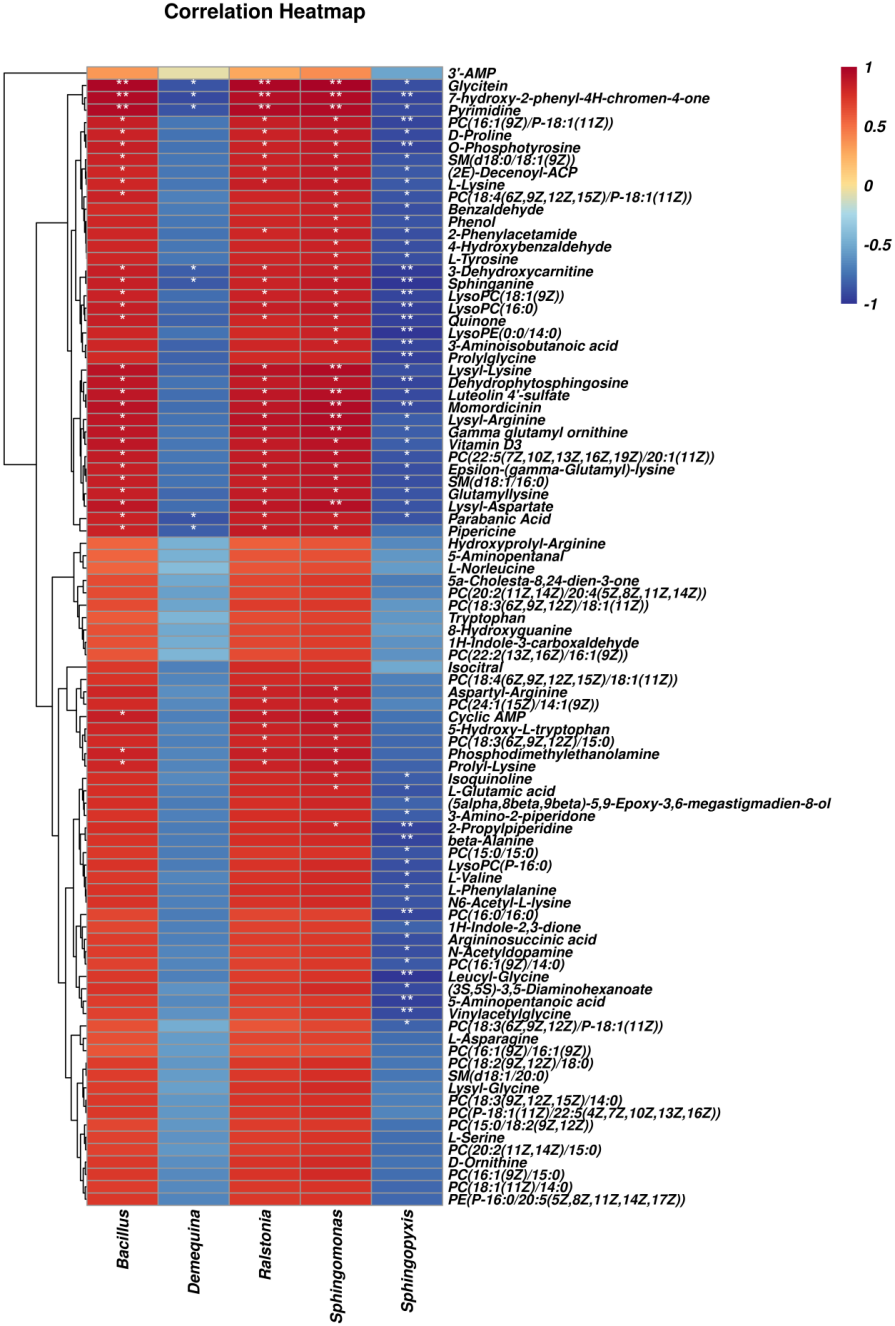
Correlation analysis of intestinal microbes and metabolites between LF and BC groups. Red represents a positive correlation; blue represents a negative–positive correlation. The darker the color, the stronger the correlation. *p < 0.05, **p < 0.01.

**Figure 8** shows significant correlations between 63 differential metabolites and five differential microbial genera found through Pearson’s correlation analysis. The correlation analysis heat map showed that *Bacillus*, *Ralstonia*, and *Sphingomonas* were positively correlated with differential metabolites and that *Demequina* and *Sphingopyxis* negatively correlated with differential metabolites. Among them, 32 metabolites were significantly positively correlated with the *Bacillus* genus, 36 with the *Ralstonia* genus, 46 with the *Sphingomonas* genus, and 57 with the *Sphingopyxis* genus, and seven were negatively associated with the *Demequina* genus.

### Correlation analysis of selected differential metabolites and intestinal genes

**Figure 9:**
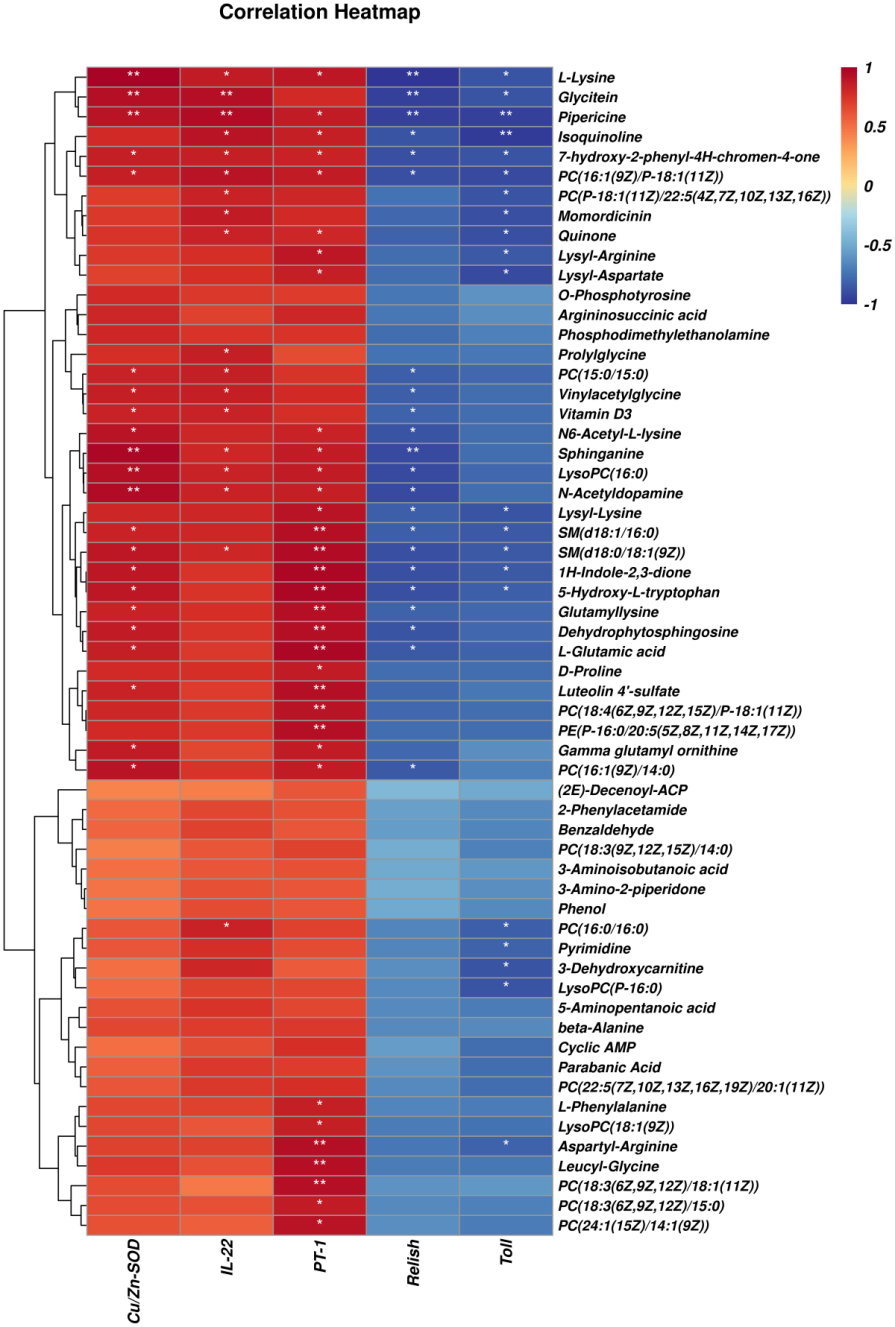
Correlation analysis of selected differential metabolites and intestinal genes between LF and BC groups. Red represents a positive correlation; blue represents a negative–positive correlation. The darker the color, the stronger the correlation. *p < 0.05, **p< 0.01.

As shown in **Figure 9**, differential metabolites with significant microbial correlation were selected for further correlation analysis with intestinal genes. The results showed that immune and intestinal barrier-related genes (*Cu/Zn-SOD*, *IL-22*, and *PT-1*) were positively correlated with differential metabolites. Inflammation-related factor (*Toll* and *Relish*) expression was negatively correlated with differential metabolite levels. Pearson’s correlation analysis revealed that the primary differential metabolites (significant correlation with more than three genes) associated with intestinal immune genes were as follows: l-Lysine, Glycitein, Pipericine, Isoquinoline, 7-Hydroxy-2-phenyl-4*H*-chromen-4-one, PC(16:1(9*Z*)/P-18:1(11*Z*)), Quinone, PC(15:0/15:0), Vinylacetylglycine, Vitamin D3, *N* ^6^-Acetyl-l-lysine, Sphinganine, LysoPC(16:0), *N*-Acetyldopamine, Lysyl-Lysine, SM(d18:1/16:0), SM(d18:0/18:1(9*Z*)), 1*H*-Indole-2,3-dione, 5-Hydroxy-l-tryptophan, Glutamyllysine, Dehydrophytosphingosine, l-Glutamic acid, and PC(16:1(9*Z*)/14:0).

## Discussion

Aquaculture relies heavily on fish meal to produce aquafeeds, leading to its continually inflated price. As a cost-effective and sustainable alternative to fish meal, various plant proteins are used to partially or entirely replace fish meal. However, an inappropriate plant protein substitution ratio could adversely affect aquatic animals’ growth due to limiting amino acids and anti-nutritional factors (6, 22). Using probiotic bacteria to alleviate the adverse effects of growth suppression in aquatic animals induced by plant protein substitution is becoming an effective auxiliary strategy (23). Similarly, in our study, 10% fish meal substitution (basal fish meal level of 25%) by CPC depressed the growth to some extent, and *B. coagulans* supplementation alleviated the downregulated levels of WGR and SGR. However, there were no significant differences in growth and survivability among these three groups. Meanwhile, the phenotypic differences in hemolymph antioxidant indicators (MDA and CAT) coincided with the growth indicators variation and trend among the groups. The significantly upregulated SOD activity reflected the improvement of the overall antioxidant capacity of *M. rosenbergii* after treatment with *B. coagulans*, which echoed the upregulated growth performance following *B. coagulans* fortification, indicating the growth promotion function of *B. coagulans* in the model of fish meal replacement by plant protein. The growth performance of animals is closely related to intestinal physiological function. The activity of digestive enzymes, the integrity of barrier structure, and the immune function of the intestine directly determine the utilization efficiency of nutrients in the feeds and the body’s defense against environmental pathogens. Also, the primary mechanism by which dietary probiotics augment the growth of cultured species is by improving intestinal digestive and absorptive activity, and health (24, 25). Given this, we further explored the effects of fish meal replacement and probiotic supplementation on intestinal digestive enzyme activity and health status.

In this study, substituting 40% fish meal with CPC considerably decreased intestinal digestive enzyme activities (trypsin and lipase). This result is consistent with previous studies that fish meal replaced with excessive plant proteins elicited intestinal villus morphology destruction and suppressed the digestive and absorptive-related enzyme activities in aquatic animals (26). The inherent anti-nutritional factors or unbalanced amino acid composition in high-plant-protein diets may be intrinsically responsible for this (27). Gamma-glutamyl transferase (γ-GT) is located on the surface of the brush border epithelium in the intestine. It is essential in the epithelial cells’ amino acid uptake and dipeptide transport (28). In addition, gene expression of γ-GT is highly sensitive to oxidative stress as part of the cellular antioxidant defensive system (29), as γ-GT is involved in the degradation and resynthesis of glutathione *in vivo* and regulates GSH levels. There is a relationship between the intestine structural damage and the upregulated level of γ-GT in the intestine. In this study, the intestine γ-GT enzyme activity was significantly upregulated following the replacement of fish meal with CPC. On the one hand, the differentiation in amino acid composition between the two diets may result in the intestinal epithelial cells’ differential amino acid absorption strategy. On the other hand, reflecting that plant protein substitution may stimulate the onset of intestinal oxidative stress. Contrary to other research results, the dietary supplementation with *B. coagulans* significantly increased the activities of the digestive enzymes, including trypsin. In this study, *B. coagulans* supplementation with high-protein plant diets significantly downregulated or somewhat did not improve to a higher level of the trypsin activities, speculating for a reason related to the timing and tissue region of sampling. The spatial and temporal orders of the digestive enzymes involved in protein and peptide digestion in aquatic animals differ (30). Our sampling node may miss the active phase of trypsin action or *B. coagulans* altered the strategy for protein digestion and absorption in the intestine. However, the exact reasons need to be further explored.

In addition to digestive and absorptive capacity, the barrier and immune functions of the gut are also crucial to the health of aquatic animals. To investigate the effects on intestinal barrier function and inflammatory response in *M. rosenbergii*, we evaluated the variations in gene expression levels of the gut barrier protein *PT-1*, inflammatory-related factors (*Relish*, *Toll*, and *IL-22*), and antioxidant factor (*Cu/Zn-SOD*). In invertebrates, the peritrophic membrane (PM) protects the epithelium cells from mechanical abrasion and prevents pathogens from adhering to intestine epithelial cells (31). Moreover, the dominant proteins of PMs were identified mainly including digestion-related, immune-related, antioxidant proteins, and structural proteins of PM in *Litopenaeus vannamei*, highlighting its essential role in the intestinal immune system (32). Peritrophins (PTs) are essential for the generation of PM, which directly affect the permeability, elasticity, porosity, and strength of PM that lines the crustacean midgut. Therefore, PT plays a role in the immune response against pathogenic bacteria by protecting the intestine from colonization, intrusion, and secondary tissue distribution of pathogenic bacteria in crustaceans (33). The expression of intestinal inflammatory transcription factors (*Toll* and *Relish*) was significantly upregulated, while the expression of barrier factor *PT-1* was significantly downregulated following the substitution of 40% fish meal by cottonseed protein concentrate, as determined. This indicates that plant protein substitution caused impairment of intestinal barrier function and secondary inflammatory responses. This may be attributed to anti-nutritional factors in plant proteins, which could impair the intestine peritrophic membrane’s morphological structure and thus induce gut inflammation (significantly upregulated inflammation factor) (34). Interleukin (IL)-22 is a homeostatic cytokine that promotes the expression of a series of genes that enforce intestinal epithelial cell defense function (35). For example, IL-22 signaling promoted the upregulation of intestine membrane mucin Muc17 in neonatal mice, suggesting its critical role in the epithelial barrier function of the intestine (36). Moreover, the reparative cytokine IL-22 was confirmed to drive repair in the intestinal epithelium through decreasing inflammation response in the colitis model (37). Additional supplementation with *B. coagulans* significantly alleviated the upregulated expression of inflammatory factors. Meanwhile, it significantly upregulated the expression levels of the barrier factor *PT-1*, the reparative cytokine *IL-22*, and the antioxidant factor *Cu/Zn-SOD*. The above results indicate that *B. coagulans* can alleviate the side effects of plant protein substitution and that the critical target regulatory molecules were *PT-1* and *IL-22*. Similar studies have shown that probiotic *Lactobacillus casei* treatment strengthened the function of the gut mucosal epithelium by boosting *IL-22* and tight junction protein (*Zonulin-1* and *Claudin-1*) expression levels in the intestinal inflammatory model of chicks infected with *Salmonella pullorum* (38).

From the gut microbiota perspective, partial substitution of fish meal with plant protein sources affected the composition and diversity of gut microbes, and inappropriate substitution ratio induced host intestine mucosa lesions and hypoimmunity (26, 39). At the phylum level, consistent with the previous study, Proteobacteria, Firmicutes, Bacteroidetes, and Tenericutes were determined as the predominant phyla in *M. rosenbergii* intestine (40). We noted a remarkable downregulation of Firmicutes abundance after plant protein substitution. Speculating that the fiber content in the CPC substitution group diet is higher than the HF group, a recent report suggested that dietary fiber intake decreases the proportion of Firmicutes (41). The Firmicutes phylum includes the vast majority of lactic acid bacteria genera, such as *Streptococcus*, *Lactobacillus*, *Leuconostoc*, and *Carnobacterium*, which are generally considered to be probiotics for intestinal health (42). The phylum Firmicutes produces short-chain fatty acids (SCFAs) through collaboration with oligosaccharide fermentation bacteria such as Bifidobacteria (43). Thus, the callback of Firmicutes abundance after *B. coagulans* intervention suggests that *B. coagulans* has the potential to improve the structure of the intestinal flora positively. In addition, substituting 40% fish meal with CPC significantly decreased the richness and diversity of the intestinal flora, as well as downregulated the abundance of *Flavobacterium* and *Bacillus*, which were regarded as antagonistic probiotics in aquaculture (44). Furthermore, the potential pathogenicity in the CPC substitution group apparently increased through the phenotype prediction of potentially_pathogenic by BugBase software. This indicated that this substitution ratio led to the unbalance of a game between probiotics and conditionally pathogenic bacteria, disrupting intestinal health. This is consistent with previous studies showing that appropriate levels of CPC substitution can increase the richness and diversity of gut flora, while excessive substitution induces enteritis and disrupt the homeostasis and part of the functions of the gut microbiota (45, 46). In recent years, *Bacillus* spp. has been reported as a potential probiotic candidate in aquatic farming sectors due to its high antagonistic activities and extracellular enzyme synthesis (47). Dietary *Bacillus* supplementation benefits the host’s growth, intestinal microbial homeostasis, stress resistance, and immune and anti-inflammatory response (48–51). *Sphingomonas* is also considered to be a potential probiotic for aquaculture. The prebiotic effects mainly include the following ways: 1) regulates nutrient metabolism by secreting cellulase, protease, and amylase (52); 2) degrades toxic compounds, ammonia nitrogen, and nitrite (53); 3) produces oligosaccharide prebiotics (54); 4) inhibit *Vibrio* (53, 55)). In this study, we noted that *B. coagulans* treatment significantly improved the relative abundance of *Sphingomonas*, *Bacillus*, and *Ralstonia* while reducing the potential pathogenic flora after CPC substitution, echoing the trend in gene expression of intestinal inflammatory and barrier factors. This indicates that *B. coagulans* treatment is conducive to the colonization of *Bacillus* spp. in the gut and has a positive regulatory effect on intestinal immunity. This is similar to a recent study on turbot that different species of *Bacillus* treatment induced a significant positive correlation between the immune and antioxidant indexes and intestinal probiotics such as *Bacillus*, *Ralstonia*, and *Bifidobacterium* and a negative correlation with pathogenic bacteria (50).

Strikingly, a new research perspective has been condensed through integrating clinical monitoring, 16S rDNA and metagenomic sequencing, metabonomics analysis, and whole-genome sequencing. Accumulating medical reports have revealed the decisive role of specific gut microbes and gut microbes-derived secondary metabolites in metabolic and gastrointestinal disease (insulin resistance, non-alcoholic fatty liver, and chronic enteritis) (56–58). Studies likewise highlighted that the secondary metabolites of intestinal microorganisms, for instance, secondary bile acids, short-chain fatty acids, and branched-chain amino acids, play a substantial role in the process of medicine and diet treatment-induced remission (13, 59). These findings raised our focus on gut microbiota-derived metabolites. In this study, we identified several differential metabolites through metabonomics analysis, mainly sphingolipids, amino acids, amino acid derivatives, phospholipids, etc. Sphingolipids are a class of lipids with a long chain of amino alcohol sphingolipid backbones and amide-bound fatty acyl chains. As signaling molecules, sphingolipids mediated microbial-host interactions, such as immunity and inflammation. Sphingolipids have been identified as the most differential biomarker metabolites in the stool of inflammatory bowel disease patients (60). Known sphingolipid-producing bacteria include most of the Bacteroidetes phylum, a few members of the Chlorobi phylum, and a subset of α-Proteobacteria (*Sphingomonas*, *Acetobacter*, and *Neospora*). Sphingolipid metabolites are essential signaling molecules mediating host cell processes, such as ceramide, sphingosine, and sphingosine-1-phosphate (S1P) (61). Sphingosine and dehydrophytosphingosine are the precursors of S1P, which play critical immunological functions in intestinal tissue (62). Our study identified the increased sphingosine and dihydrosphingosine, and the upregulated abundance of sphingosine-producing bacteria *Sphingomonas* after *B. coagulans* intervention. Meanwhile, Sphingolipid metabolism-related pathways (Sphingolipid signaling pathway and Sphingolipid metabolism) were enriched. In addition, correlation analysis established a significant positive correlation between sphingosine, *Sphingomonas*, and immune-related genes (*Cu/Zn-SOD*, *IL-22*, and *PT-1*) and a significant negative correlation with inflammatory factor *Relish*. The above results indicated that the *Sphingomonas*–sphingosine axis is involved in the intestinal inflammatory repair and immune regulation of *M. rosenbergii* mediated by *B. coagulans*.

**Figure 10:**
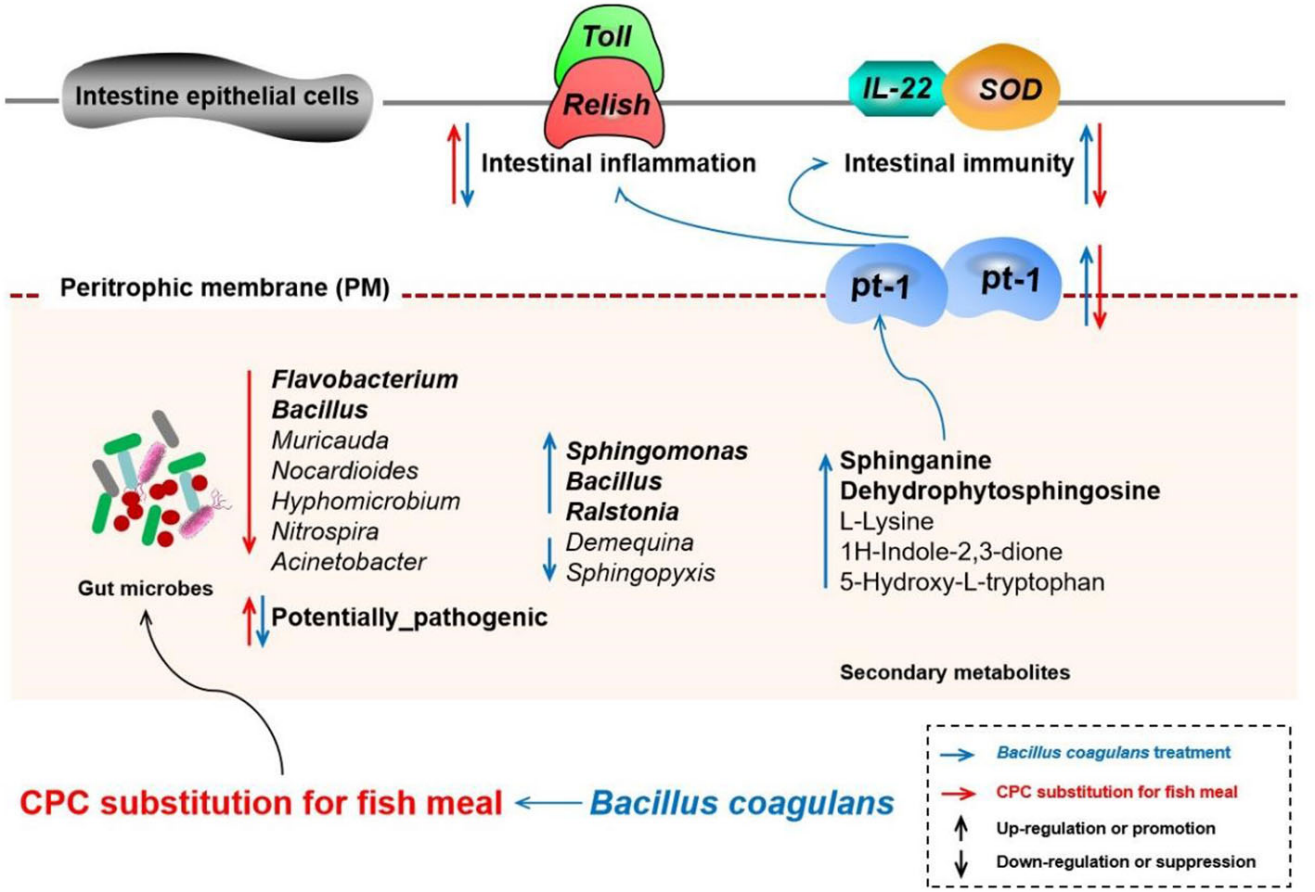
Schematic diagram depicting the effects and possible mechanisms of the effects of CPC substitution and *Bacillus coagulans* intervention on gut microbes, derived secondary metabolites, and intestinal physiological functions of *Macrobrachium rosenbergii*. The red arrow represents the action path of CPC substitution, and the blue arrow represents the action path of *Bacillus coagulans* intervention. The upward arrow represents upregulation or promotion, and the downward arrow represents downregulation or suppression. CPC, cottonseed protein concentrate.

In terms of amino acids, studies have proved that intestinal microorganisms can synthesize, incorporate, and metabolize several available amino acids to maintain amino acid homeostasis through bidirectional switching with the host. In addition, the microbially derived metabolites mediate mucosal immunoregulation, epithelial cell barrier maintenance, and enteroendocrine regulation, such as serotonin, ammonia, hydrogen sulfide, branched-chain amino acids, polyamines, and phenolic and indolic compounds (63). In mammals, l-tryptophan, endogenous tryptophan metabolites, and bacterial tryptophan metabolites-indole derivatives could enhance intestinal barrier function by promoting the expression of tight junction protein of intestinal epithelial cells (63) and regulating the expression of inflammatory-related factors (such as anti-inflammatory cytokine IL-22) (64). In addition, sufficient evidence suggested that lysine (65), glycine (66), arginine (67), etc., can shape the intestinal microbial composition and improve the host intestinal mucosal immunity. In the present study, we noted that *B. coagulans* treatment considerably affected the amino acid metabolic profile of the *M. rosenbergii* gut microbes, such as l-Lysine, Lysyl-Lysine, Epsilon-(gamma-glutamyl)-lysine, Tryptophan, 5-Hydroxy-l-tryptophan, 1*H*-Indole-2,3-dione, Lysyl-Arginine, Aspartyl-Arginine, and Leucyl-Glycine. Moreover, the correlation analysis also established a significant positive correlation between the above amino acid metabolites and the intestinal factors PT-1 and Cu/Zn-SOD. These results showed that changes in the amino acid metabolic spectrum of gut microorganisms induced by *B. coagulans* treatment are correlated with the intestinal immunity and barrier function of *M. rosenbergii*. However, to date, studies mainly focused on exogenous amino acid’s contribution to hosting intestine immunity but rarely explored the effect and mechanism of bacterial amino acid intermediates themselves on the host physiology through the intestinal epithelial cells; their contribution degree and fate remain to be further explored. In addition, our study also established the correlation between other differential metabolites (glycitein, pipericine, isoquinoline, 7-hydroxy-2-phenyl-4*H*-chromen-4-one, PC(16:1(9*Z*)/P-18:1(11*Z*)), PC(15:0/15:0), PC(16:1(9*Z*)/14:0), LysoPC(16:0), SM(d18:1/16:0), SM(d18:0/18:1(9*Z*)), Vitamin D3, and *N*-acetyldopamine) and intestinal function genes (*Cu/Zn-SOD*, *IL-22*, *PT-1*, *Toll*, and *Relish*). Our results suggested that the effects of *B. coagulans* on gut microbe metabolism are multifaceted, and the positive regulation of intestinal immunity, barrier function, and inflammatory responses in *M. rosenbergii* depends on the combined action of multiple metabolites (**Figure 10**). However, the precise mechanisms require further research.

## Data availability statement

The raw dataset for 16SrDNA sequencing have been deposited in the National Institutes of Health’s Short Read Archive database (SRA accession no. PRJNA894136; BioSample accessions: SAMN31438493, SAMN31438494, SAMN31438495, SAMN31438496, SAMN31438497, SAMN31438498, SAMN31438499, SAMN31438500, SAMN3138501).

## Author contributions

XZ contributed in the areas of experimental design, sampling, data analysis, and write-up. BL, YZ, QZ and XZ contributed to the experimental design and manuscript review. JY and NW contributed to feeding and cultivating experimental prawns, sampling, and statistics. All authors contributed to the article and approved the submitted version.

## Funding

This work was supported by the Project of National Key R&D Program of China (2019YFD0900200), China Agriculture Research System of MOF and MARA (CARS-48), Central Public-interest Scientific Institu-tion Basal Research Fund, CAFS (2020TD59).

## Conflict of interest

The authors declare that the research was conducted in the absence of any commercial or financial relationships that could be construed as a potential conflict of interest.

## Publisher’s note

All claims expressed in this article are solely those of the authors and do not necessarily represent those of their affiliated organizations, or those of the publisher, the editors and the reviewers. Any product that may be evaluated in this article, or claim that may be made by its manufacturer, is not guaranteed or endorsed by the publisher.
